# Correction: Singh, M.P., *et al.* Cytoskyrins and Cytosporones Produced by Cytospora sp. CR200: Taxonomy, Fermentation and Biological Activities

**DOI:** 10.3390/md70200095

**Published:** 2009-04-14

**Authors:** Maya P. Singh, Jeffrey E. Janso, Sean F. Brady

**Affiliations:** 1 Natural Products Research, Chemical and Screening Sciences, Wyeth Research, Pearl River, NY 10965, USA; 2 Department of Biological Chemistry and Molecular Pharmacology, Harvard Medical School, Boston, MA 02115, USA

We found an error in Figure 1 in our paper published in the Marine Drugs [1]. The structure of Cytosporones A and B are corrected as follows. Additional chemistry details of these compounds can be found in our earlier paper [2].

We apologize for any inconvenience caused to the readers.

## Figures and Tables

**Figure 1:**
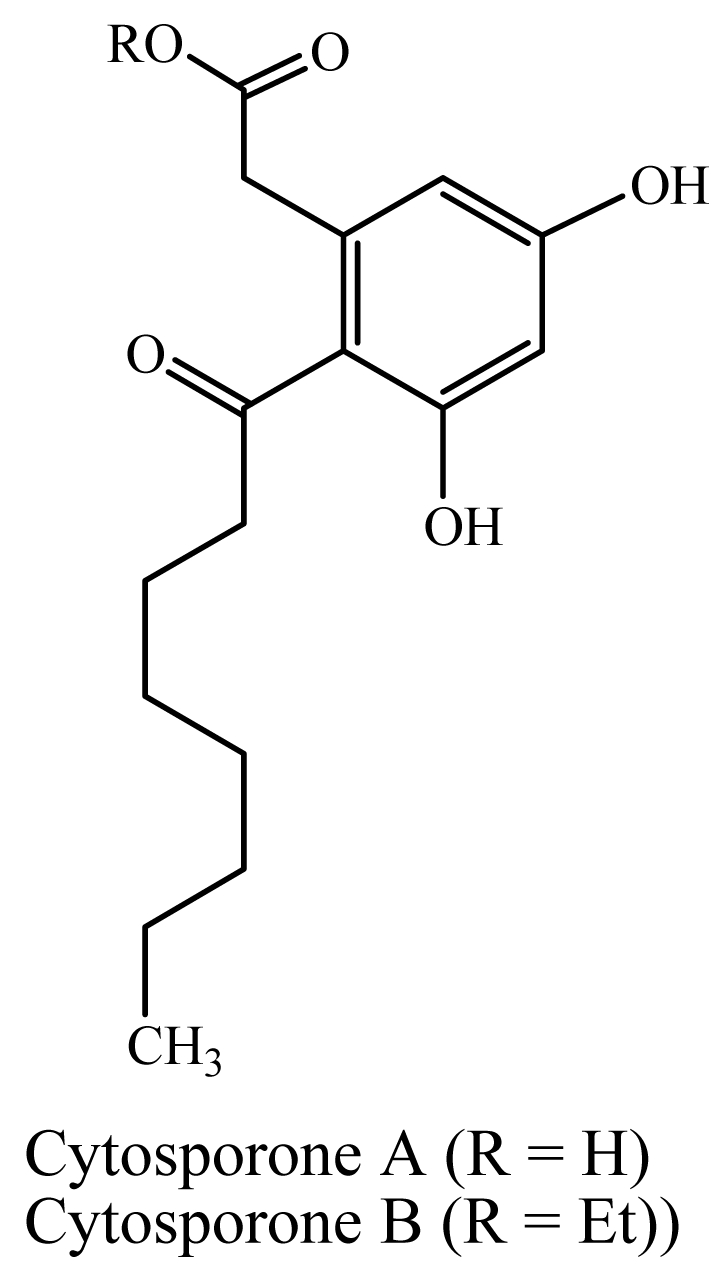
Chemical structures of cytosporones A and B in figure 1.
